# Using semantics for representing experimental protocols

**DOI:** 10.1186/s13326-017-0160-y

**Published:** 2017-11-13

**Authors:** Olga Giraldo, Alexander García, Federico López, Oscar Corcho

**Affiliations:** 1 Ontology Engineering Group, Madrid, Universidad Politécnica de Madrid, Madrid, 28660 Spain; 2 Universidad Del Valle, Cali, Colombia

**Keywords:** Semantic web, Graph theory, Ontologies, RDF for experimental protocols, Knowledge representation, Linked data

## Abstract

**Background:**

An experimental protocol is a sequence of tasks and operations executed to perform experimental research in biological and biomedical areas, e.g. biology, genetics, immunology, neurosciences, virology. Protocols often include references to equipment, reagents, descriptions of critical steps, troubleshooting and tips, as well as any other information that researchers deem important for facilitating the reusability of the protocol. Although experimental protocols are central to reproducibility, the descriptions are often cursory. There is the need for a unified framework with respect to the syntactic structure and the semantics for representing experimental protocols.

**Results:**

In this paper we present ***“SMART Protocols ontology”***, an ontology for representing experimental protocols. Our ontology represents the protocol as a workflow with domain specific knowledge embedded within a document. We also present the ***S*** *ample* ***I*** *nstrument* ***R*** *eagent* ***O*** *bjective* (SIRO) model, which represents the minimal common information shared across experimental protocols. SIRO was conceived in the same realm as the **P**atient **I**ntervention **C**omparison **O**utcome (PICO) model that supports search, retrieval and classification purposes in evidence based medicine. We evaluate our approach against a set of competency questions modeled as SPARQL queries and processed against a set of published and unpublished protocols modeled with the SP Ontology and the SIRO model. Our approach makes it possible to answer queries such as *Which protocols use tumor tissue as a sample*.

**Conclusion:**

Improving reporting structures for experimental protocols requires collective efforts from authors, peer reviewers, editors and funding bodies. The SP Ontology is a contribution towards this goal. We build upon previous experiences and bringing together the view of researchers managing protocols in their laboratory work. Website: https://smartprotocols.github.io/.

## Background

Experimental protocols are fundamental information structures that support the description of the processes by means of which results are generated in experimental research [1]. Experimental protocols describe how the data were produced, the steps undertaken and conditions under which these steps were carried out. Biomedical experiments often rely on sophisticated laboratory protocols, comprising hundreds of individual steps; for instance, the protocol for chromatin immunoprecipitation on a microarray (Chip-chip) has 90 steps and uses over 30 reagents and 10 different devices [2]. Nowadays, such protocols are generally written in natural language and presented in a “*recipe*” style, so as to make it possible for researchers to reproduce the experiments.

The quality of experimental protocols reported in articles is a cause of concern. Reproducibility, central to research, depends on well-structured and accurately described protocols. Kilkenny et al. [3] found that 4 percent of the 271 journal articles assessed did not report the number of animals used anywhere in the methods or the results sections. Assessing statistical significance requires to know the number of animals participating in an experiment; it is also necessary if the experimental methods are to be reproducible, reused and adapted to similar settings. High-quality description of experimental methods is also critical when comparing results and integrating data. In an effort to address the problem of inadequate methodological reporting, journals such as Nature Protocols [4], Plant Methods (Methodology) [5] and Cold Spring Harbor Protocols [6], have guidelines for authors that include recommendations about the information that should be documented in the protocols. The ISA-TAB also illustrates work in this area; it delivers metadata standards to facilitate data collection, management and reuse from “*omic-based*” experiments [7]. The BRIDG initiative [8] aims to formalize a shared view of the dynamic and static semantics of protocol-driven research. The BioSharing initiative [9], is a catalog of standards promoting the representation of information in the life, environmental and biomedical sciences [9]. STAR [10] is an effort that proposes to “*Empowering Methods*” offering a overview of resources used in a study. Ontologies such as EXACT [11, 12] aim to formalize the description of protocols focusing on experimental actions; the BioAssay Ontology (BAO) [13] describes biological screening assays and their results; the eagle-i resource ontology (ERO) [14] represents some aspects related to protocols.

Here we present SMART Protocols ontology (henceforth SP), our ontology for representing experimental protocols; we aim to *“facilitate the semantic representation of experimental protocols”*. Our representation makes it possible to answer queries such as *“Which protocols use “tumor tissue” as a sample?”*, *“Retrieve the reagents and the corresponding information from the manufacturers for a specific protocol”*, *“retrieve the diseases caused by the reagents used in a specific protocol”*. These and other queries can be processed at our SPARQL endpoint ^1^. The SP Ontology provides the structure and semantics for data elements common across experimental protocols. For representing reagents, samples, instruments and experimental actions we reuse ontologies such as the Chemical Entities of Biological Interest (ChEBI) [15], NCBI taxonomy [16–18], the Ontology for Biomedical Investigations (OBI) [19], the BioAssay Ontology (BAO), The Experimental Factor Ontology (EFO) [20], eagle-i resource ontology (ERO), Cell Line Ontology (CLO) [21, 22], and EXACT. We also reuse and extend classes from the Information Artifact Ontology (IAO) [23]. In this paper we also present the SIRO model; this is a minimal information model for the representation of ***S*** *amples* ***I*** *nstruments* ***R*** *eagents* ***O*** *bjective* (hence SIRO). This model has been conceived in a way similar to that of the **P**atient **I**ntervention **C**omparison **O**utcome (PICO) model; it helps to frame questions and provides an anchor for the records [24]. SIRO facilitates classification and retrieval without exposing the content of the document. In this way, publishers and laboratories may keep the content private, exposing only the information that describes the sample, instruments, reagent and objective of the protocol. As an illustration, in this paper we use the protocol “*Extraction of total RNA from fresh/frozen tissue (FT)*” [25] as a running example. We represent this protocol with the SP ontology and SIRO.

## Methods

**Fig. 1:**
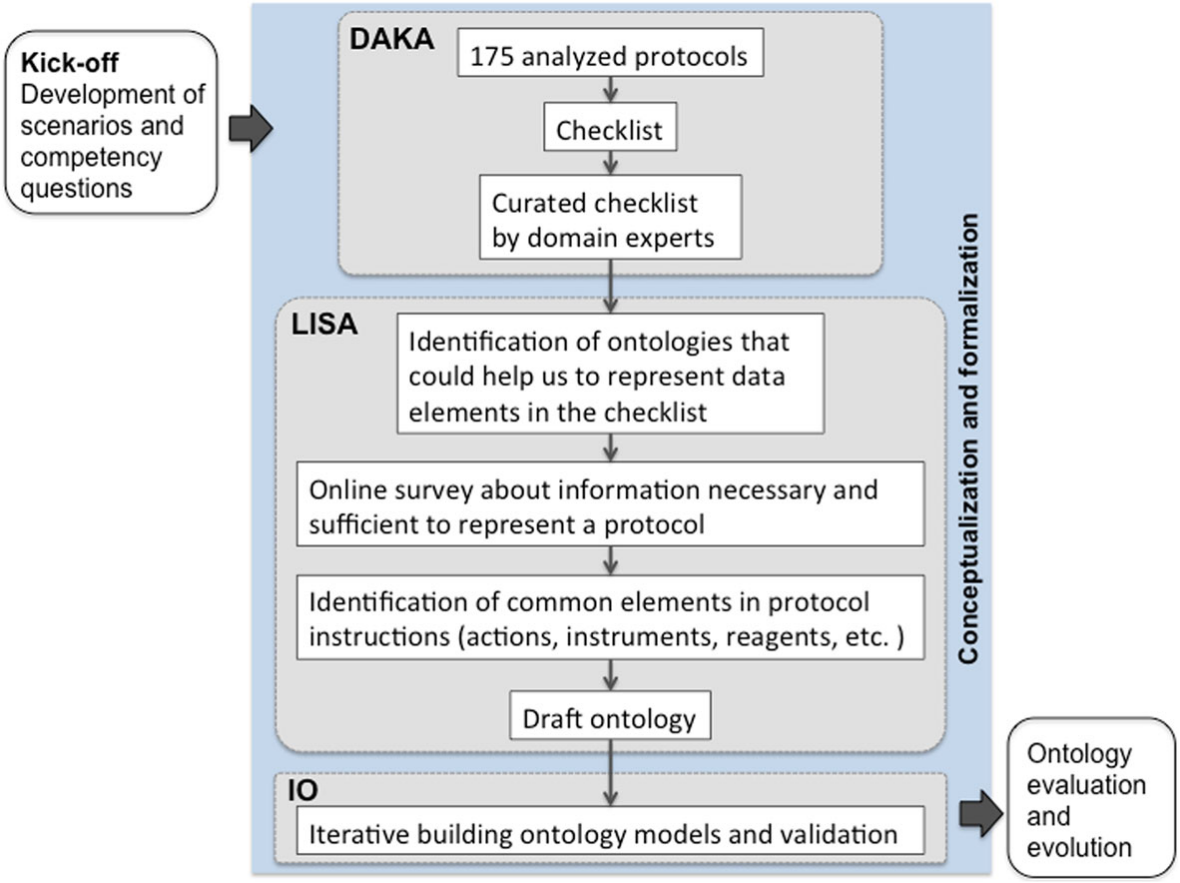
Developing the SMART Protocols ontology, methodology

Our SMART Protocols ontology [26] is based on an exhaustive analysis of 175 published and unpublished experimental protocols (see Table 1 in Domain Analysis and Knowledge Acquisition, DAKA); we also analyzed on-line repositories and guidelines for authors. For the development of the SP Ontology [1] we have followed the practices recommended by the NeOn methodology [27], as well as those reported by García [28]. For example, we used conceptual maps to better understand the correspondences, relations and possible hierarchies in the knowledge we were representing. The stages and activities we implemented throughout our ontology development process are illustrated in Fig. 1 and explained below. For the ontology development process we also considered the guidelines from the OBO foundry [29].

**Table 1:** Repositories and number of protocols analyzed

Repository	Bio Tech	CSH	CP	GMR	JoVE	NPE	PM	PO	SP	CIAT
No. of protocols	6	9	25	5	21	13	12	5	4	75
Total	175									

The protocols are available at: https://smartprotocols.github.io/

### The kick-off, scenarios and competency questions

In the first stage, we gathered motivating scenarios, competency questions, and requirements. We focused on the functional aspects that we wanted the ontology to represent. Domain experts were asked to provide us with a list of competency questions, these are presented in our website^2^. Some of the competency questions we gathered include, “retrieve the protocols using a given sample” and, “which protocols can I use to process this sample given that I only have X and Z reagents”. Competency questions were initially used to scope the domain for which we were developing the ontology; these questions were also used during the evaluation.

### Conceptualization and formalization

In this stage we identified reusable terminology from other ontologies; for supporting activities throughout this stage we used BioPortal [30] and Ontobee [31]. We also looked into minimal information standards [32], guidelines and vocabularies representing research activities [33–35]. Issues about axioms required to represent this domain were discussed and tested in Protégé v. 4.3 and 5.0 [36]; during the iterative ontology building, classes and properties were constantly changing. We identified, and explain below, three main activities throughout this stage, namely: Domain Analysis and Knowledge Acquisition (DAKA), Linguistic and Semantic Analysis (LISA), Iterative ontology building and validation (IO).

#### Domain analysis and knowledge acquisition, DAKA

We manually reviewed 175 published and unpublished protocols from topic areas such as molecular biology, cell and developmental biology, biochemistry, biotechnology, microbiology and virology, as well as guidelines for authors from journals. The unpublished protocols (75 in total) were collected from four laboratories located at The International Center for Tropical Agriculture (CIAT) [37]. The published protocols (open access) were gathered from 9 repositories; Table 1 presents the list of journals and the number of protocols that we analyzed. We used these sources to prepare a checklist with data elements that were required in guidelines for authors and also present in published protocols –see Annex 1^3^. This was the seed for our discussions with domain experts.

Our domain analysis focused on gathering terminology and data elements, idem higher abstractions that could be used to group terminology. Domain experts were bringing their protocols and discussing specific issues, e.g. what was missing for applying a particular protocol. As the discussions were progressing, published and unpublished protocols were added to the mix. Due to time constraints domain experts were not required to work before or after the workshops. Olga Giraldo was the facilitator for the DAKA activities. This made the processes with domain experts more efficient because she has extensive experience in laboratory practices. Ten domain experts participated in DAKA; they all had hands-on experience in areas such as molecular biology, virology, plant breeding, biochemistry, clinical microbiology and pathology. From DAKA we confirmed most of the data elements in our initial checklist and identified clusters of terminology, e.g. samples and instruments. The output of this activity was an improved checklist and relations to the information in the protocols. This output was used as input for the linguistic analysis.

#### Linguistic and semantic analysis, LISA

From our corpus of protocols we selected 100 documents; these represented the topic areas for which we had domain experts. We tried to have some complex and lengthy protocols involving several procedures and technologies; for instance, protocols describing the development of an SNP genotyping resource [38] and protocols describing the construction of an RNA-seq library [39]. We also worked with simpler protocols such as sample preparation or DNA extraction protocols. The terminology gathered in DAKA was discussed with domain experts and analyzed against existing ontologies; BioPortal and Ontobee were used to browse the ontologies in order to determine how terms were related to biomedical ontologies and which were the ontologies that could be relevant for this work.

Throughout this activity we also addressed the representation of workflows in the protocols. This was particularly problematic because domain experts did not agree on how granular the descriptions of the workflows and the relation between steps needed to be, how to indicate order in the sequence of operations and, what information was obligatory in the description of the steps. In this activity we used an on-line survey that helped us to determine and validate what data elements were necessary and sufficient for the description of the protocols –see Annex 2^3^. We used the outputs from DAKA in the survey and asked participants to indicate whether a particular data element was relevant or not; an invitation to participate was circulated over mailing lists, participants did not have to disclose their identity. Twenty participants filled up the survey; this survey helped us to informally validate the outputs from DAKA and also gave us another perspective about relevant data elements in the description of protocols. Results from the survey are available in Annex 3^3^.

From this activity, we identified linguistic structures that authors were using to represent actions. We were interested in understanding how verbs were representing actions and what additional information was indicating the attributes for actions. For instance, “*Fresh-leaf tissue (0.2 g) was* ***ground*** *in a 1.5-mL Eppendorf tube with a* ***micropestle*** *and preheated freshly prepared 800 uL* ***extraction buffer*** *was immediately added to the tube”* [40] is a commonly used cell disruption step in nucleic acids and protein extraction protocols. In our corpus of documents, these steps were usually described using verbs like “break, chop, grind, homogenize”. There are also common methods for specific operations; for instance, for breaking the cells the methods were “blending, grinding or sonicating” the sample. The sequence of instructions had an implicit order that was not always clearly specified as authors sometimes hide it in the narrative. There is, however, an input-output structure. Actions in the workflow of instructions are usually indicated by verbs; accurate information for implementing the action implicit in the verb was not always available. For instance, structures such as “Mix thoroughly at room temperature”, “Briefly spin the racked tubes” are common in our dataset. The instructions always have actions and participants, which may be samples, reagents, instruments and/or measures. This was particularly useful in the definition of our workflow; the pattern that emerged is discussed in the “Results” section. In this activity we also identified document-related data elements; for instance, roles for authors, e.g. validator, statistical reviewer. We also identified the ontologies that could represent the concepts we were working with. A draft ontology with the seminal terminology and initial classification was the output from LISA; this output was further refined during the iterative ontology building stage.

#### Iterative ontology building and validation, IO

The draft ontology from LISA was incrementally growing in complexity, number of concepts and relations. The knowledge engineer conducted continuous evaluations of the draft ontologies against competency questions. The ontology models were shared with domain experts, they reviewed the drafts, gave feedback and the ontology was updated.

As we were building ontology models, we identified the modularity needed to represent experimental protocols. From our models, we conceptualized the protocols as workflows embedded within documents. Thus, the document module of SP ontology (henceforth SP-Document) was designed to provide a structured vocabulary that could represent information for reporting an experimental protocol. The workflow module of SP ontology (henceforth SP-workflow) delivers a structured vocabulary to represent the sequence of actions in the execution of experimental protocols. The main outcome from this activity was an ontology with the SP-Document and SP-workflow modules and their corresponding classes and object properties. Our ontologies were developed using OWL-DL. We used the Protégé editor versions 4.X and 5; the Protégé plug-in OWLViz [41] was used to visualize the model.

### Ontology evaluation

during the evaluation process, we addressed issues related to the syntax, the conceptualization and formalization. We also verified whether the competency questions could be resolved by representing experimental protocols using the ontology and having the resulting RDF in a SPARQL endpoint.

We evaluated the syntax of the ontology using The OntOlogy Pitfall Scanner (OOPS) [42]; it was useful to detect and correct anomalies or pitfalls in our ontologies [43]. For instance, the identification of incomplete inverse object properties, lack of domain and range, missing annotations and issues in naming conventions. The resulting ontology from the “Conceptualization and Formalization” phase was evaluated by 10 domain experts. They were asked to determine if the proposed classes in the ontology could represent the information from a set of 13 protocols that we selected for this purpose. A list of the protocols as well as results from this evaluation are presented in Annex 4^3^.

We also tested the capability of the SMART Protocols ontology to answer the competency questions specified by domain experts; does the ontology represent enough information to answer these types of questions? do the answers require a particular level of detail or representation of a particular area? This part of the evaluation entailed the transformation of 10 experimental protocols to RDF^4^. These were uploaded in our SPARQL endpoint and the queries were formalized in SPARQL; a complete list of SPARQL queries has been made available^2^.

## Results

### The SMART protocols ontology

Our ontology reuses BFO; we are also reusing the ontology of relations (RO) [44] to characterize concepts. In addition, each term in the SP ontology is represented with annotation properties imported from OBI Minimal metadata [45]. The classes, properties and individuals are represented by their respective labels to facilitate readability. The prefix indicates the provenance for each term; for instance, the prefix sp is used to identify classes and object properties from SP ontology. For the object properties we are using italics, words or phrases representing instances are in between quotation marks, e.g. “RNA extraction”, instance of the class sp:lab procedure 3. In this section we use the protocol “*Extraction of total RNA from fresh/frozen tissue (FT)*” [25] as a running example to represents the document and workflow aspects of a protocol. Our ontology is available in BioPortal^5^, github ^6^ and also is registered at vocab.linkeddata.es^7^. vocab.linkeddata.es is a list of vocabularies developed by the Ontology Engineering Group (OEG). A graphical illustration of the ontology can be found at Annex 5^3^.

#### The document module

**Fig. 2:**
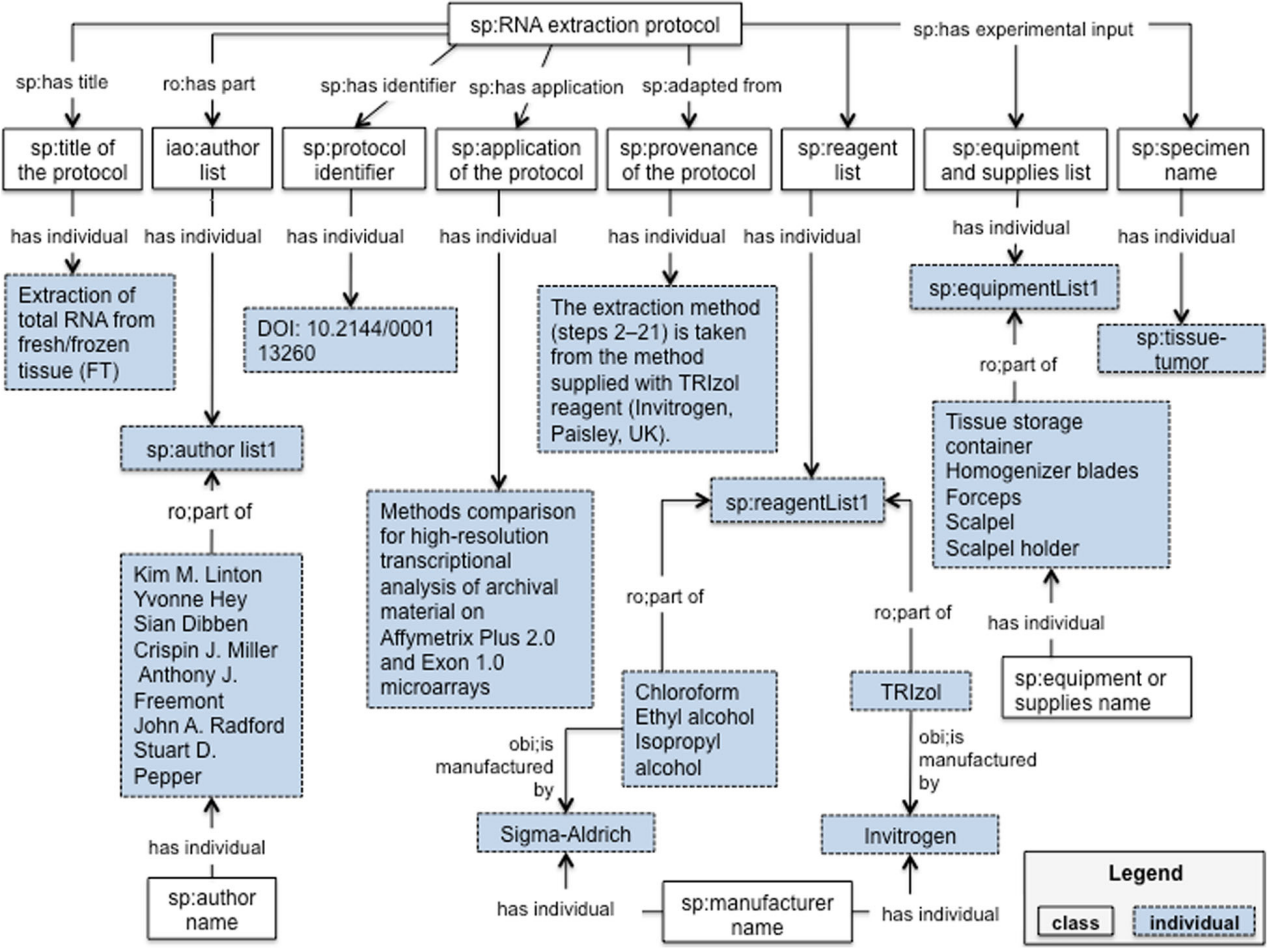
SP-Document module. This diagram illustrates the metadata elements described in Table 2. The classes, properties and individuals are represented by their respective labels

The document module of the SP ontology [46] aims to provide a structured vocabulary of terms to represent information for reporting an experimental protocol. The class iao:information content entity and its subclasses iao:document, iao:document part, iao:textual entity and iao:data set were imported from IAO. This module represents metadata elements as classes, some of them are: sp:title of the protocol, sp:purpose of the protocol, sp:application of the protocol, sp:reage- nt list, sp:equipment and supplies list, sp:manufacturer, sp:catalog number and sp: storage conditions. We have used the SP-Document modeule to represent our running example, the results are presented in Table 2 and Fig. 2; metadata elements are organized in SP-Document as information content entities. In order to facilitate the use of identifiers for the material entities like reagents and equipments, we created the object property *sp:has catalog number* and the class sp:catalog number. In this way a relation is established between the reagent or equipment and the corresponding manufacturer.

**Table 2:** Metadata represented in SP-Document

Bibliographic metadata			
sp:title of the protocol	Extraction of total RNA from fresh/frozen tissue (FT)		
sp:author name	“Kim M. Linton”, “Yvonne Hey”, “Sian Dibben”, “Crispin J. Miller”, “Anthony J. Freemont”, “John A. Radford”,		
	and “Stuart D. Pepper”		
sp:protocol identifier	DOI:10.2144/000113260		
Descriptive metadata			
sp:application of the protocol	“Methods comparison for high-resolution transcriptional analysis of archival material on Affymetrix Plus 2.0 and Exon		
	1.0 microarrays”		
sp:provenance of the protocol	“The extraction method (steps 2–21) is taken from the method supplied with TRIzol reagent Invitrogen, Paisley, UK).”		
Metadata about the materials			
sp:specimen name	“tumor tissue”		
sp:reagent name	“TRIzol”, “Chloroform”, “Ethyl alcohol”, “Isopropyl alcohol”		
sp:manufacturer name	“Invitrogen”, “Sigma-Aldrich”		
sp:equipment or supplies name	“Tissue storage container”, “Homogenizer blades”, “Forceps”, “Scalpel”, “Scalpel holder”		

#### The workflow module

**Fig. 3:**
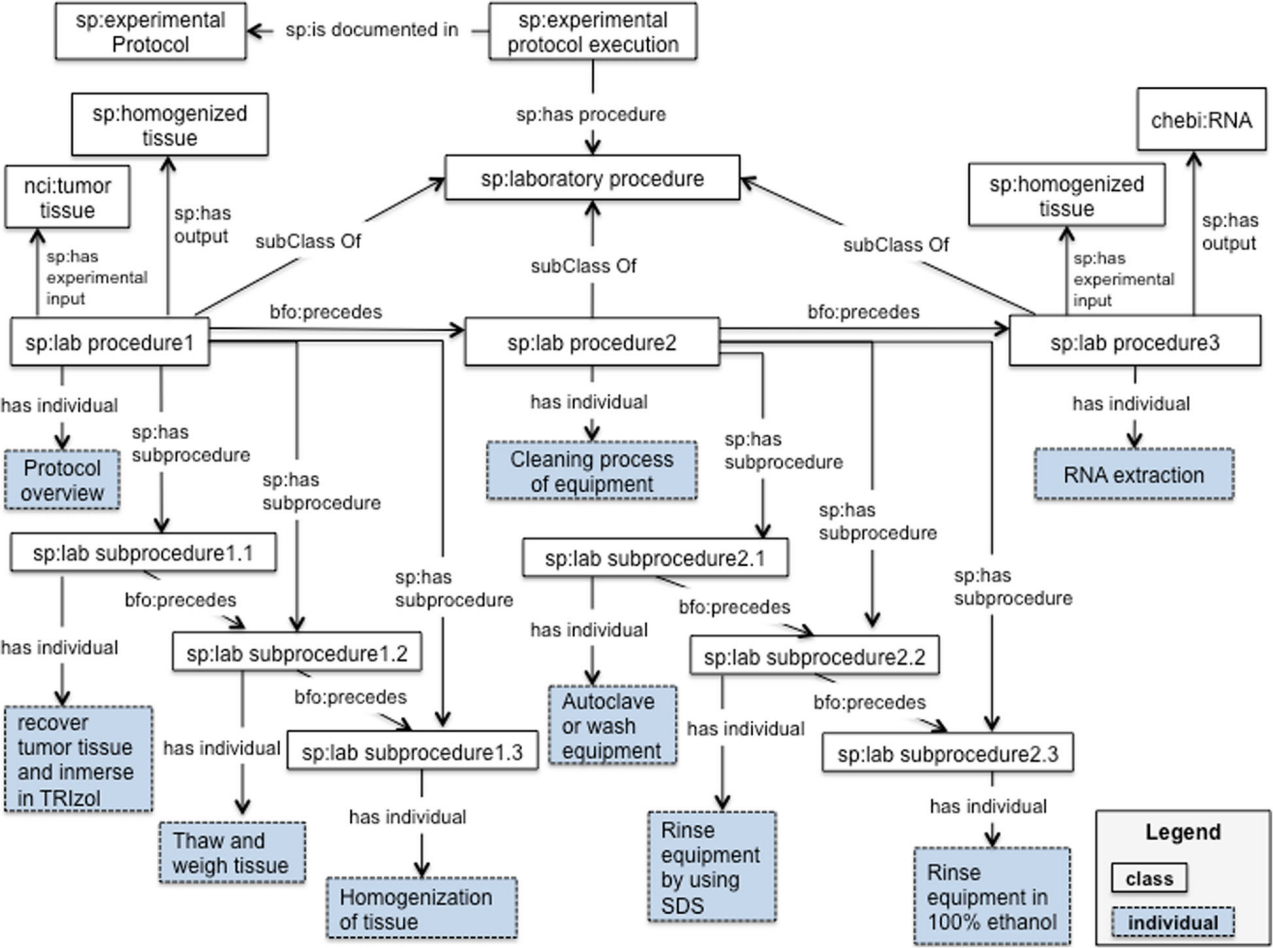
SP-Workflow module. This diagram illustrates the metadata elements described in Table 3. The classes, properties and individuals are represented by their respective labels

The SP ontology also considers the protocol as an executable element to be carried out and maintained by humans. The workflow module [47] is a descriptive model for workflows; it is not a workflow programming language. The workflow module represents the procedures, subprocedures, actions (or verbs), experimental inputs (samples/specimens) and other participants such as reagents and instruments. Experimental protocols often include a set of laboratory procedures; these transform inputs into outputs. Our running example (see Fig. 3 and Table 3), includes 3 laboratory procedures: sp:lab procedure 1 (“Protocol overview”, indicating how to process the sample), sp:lab procedure 2 (“Prior to RNA extraction: cleaning process of equipment”) and sp:lab procedure 3 (“RNA extraction”). The first column in Table 3 includes the procedures from our running example. The second column includes subprocedures or instructions for each procedure.

**Table 3:** Procedures and subprocedures from “Extraction of total RNA from fresh/frozen tissue (FT)”

Procedure	Subprocedure
Protocol overview (sp:lab procedure 1)	Recover tumor tissue at the time of surgery, trim into 1-cm3 fragments, and immerse immediately in TRIzol reagent prior to freezing at −80°.
	Thaw and weigh tissue prior to RNA extraction, working quickly.
	Use a tissue power homogenizer (or a mortar and pestle) to homogenize tissue by hand.
Prior to RNA extraction: cleaning process of equipment (sp:lab procedure 2)	Autoclave or wash equipment (i.e., tissue storage container, homogenizer blades, forceps, scalpel holder) in Neutracon solution for 2–4 h.
	Rinse equipment well in 1% SDS (prepared using DEPC-treated or other nuclease-free water).
	Rinse in 100% ethanol and leave to air-dry.
RNA extraction (sp:lab procedure 3)	Homogenize sample using tissue homogenizer.
	Add 0.2 mL chloroform per 1 mL TRIzol and cap tube tightly.
	Add 0.5 mL isopropyl alcohol per 1 mL TRIzol.
	Add 1 mL 75% ethanol per 1 mL TRIzol and vortex for 10 s.

The class sp:lab procedure 1 (“Protocol overview”) has a tumor tissue (nci:tumor tissue) as an input (*sp:has experimental input*); in a similar way, the lab procedure 1 has a homogenized tissue (sp:homogenized tissue) as an output (*sp:has output*). The laboratory procedure 1 includes 3 subprocedures (or steps/instructions) indicating how to manipulate and prepare the sample, namely: sp:lab subprocedure 1.1, sp:lab subprocedure 1.2 and sp:lab subprocedure 1.3. The order in which these subprocedures should be executed is represented by the BFO property *is preceded by* and *precedes*. The class sp:lab procedure 2 (“Prior to RNA extraction: cleaning process of equipment”) is a recipe describing how to clean the equipment to be used during the RNA extraction protocol. This recipe includes 3 steps, sp:lab subprocedure2.1, sp:lab subprocedure 2.2 and sp:lab subprocedure 2.3.

The class sp:lab procedure 3 (“RNA extraction”) has the homogenized tissue (output from the lab procedure 1) as an input and, the class chebi:RNA as an output. It includes 20 subprocedures, these are not represented in the Fig. 3 due to lack of space. We propose the classes sp:laboratory procedure and sp:laboratory subprocedure for the representation of procedures and subprocedures. The object property, *sp:has procedure*, is used to characterize the laboratory procedures that are part of the execution of an experimental protocol (sp:experimental protocol execution); the object property *sp:has subprocedure*, is used to characterize the subprocedures that are part of a given procedure. Procedures have inputs and outputs, subprocedures have participants. For cases where authors only have an extensive list of steps, the SP ontology considers these as subprocedures under a procedure container. In this way we are representing protocols with only a long list of steps as well as those with groups of steps. This also allows us to represent more complex protocols that usually result from merging several protocols.

We are representing antibodies, cell lines and plasmids as material entities. We are using *ro:derives from* to indicate that it derives from an organism; similarly, we are using the *obi:has_role* to indicate the role that it plays, as understood by the author of the protocol.

### Evaluation

#### Syntax

OOPS allowed us to identify the lack of domain and range in the object properties *ro:part_of* and ro:has_part; these were imported from the Relations Ontology (RO). We verified in the original ontology and these two properties do not have domain and range [48]. OOPS was useful for verifying the syntax of the ontology.

#### Conceptualization and formalization

The resulting ontology was evaluated by 10 domain experts, they were asked to determine whether the resulting ontology was representing the information items from experimental protocols. This evaluation was satisfactory because the information from the protocols was represented in the ontology. Interestingly, resulting from this evaluation we could identify some issues related to the way published and unpublished protocols were described. For instance, published protocols don’t have any information that facilitates the identification of roles; for instance, who is the chief scientist, who did the statistical validation, who was the lab scientist, etc. Identifying these roles was considered as important because it is an indication of quality control in the development of the protocol; this data element was identifiable in unpublished protocols and it is part of our ontology. Unpublished protocols usually have version information, as well as a short description of the roles played by those who are using, developing, standardizing or modifying the protocols.

From this evaluation it was also evident that published protocols were not consistent in the data elements that they use to represent the experimental protocol. For instance, some of the protocols had an explicit description of “advantages” and “application of the protocol”, while some others did not provide this information. A similar situation was found with respect to information about limitations. The bibliographic metadata that was identified includes, title, author, subject area and protocols identifiers (IDs). These were not always available; in the case of unpublished protocols the ID was sometimes an internal code. Although the class author identifier (sp:author identifier) could not be instantiated, we decided to leave it in the ontology because it was deemed important. Published and unpublished protocols have authors as literal values without any relation to IDs.

Published and unpublished protocols often report the name of the materials but not the manufacturer and the corresponding identifier, this is usually the catalog number. This information is frequently available and it is always necessary when trying to reuse a protocol, the SP Ontology models these data elements. Alert messages, hints, pause points, cautions or troubleshooting were represented in SMART Protocols ontology and validated by the domain experts. Although the description of the work steps, procedures, subprocedures and recipes varied across the protocols, the data elements describing the workflow could be easily represented in our ontology.

We also asked domain experts to instantiate the classes with text from the protocols. They were selecting excerpts of text and assigning classes to these narratives, e.g. “This is a simple protocol for isolating genomic DNA from fresh plant tissues” was classified as an objective, “DNA from this experiment can be used for all kinds of genetics studies, including genotyping and mapping” was classified as an application. They were also selecting some specific words and classifying them; for instance, “Isopropanol” was classified as a reagent, “mortar and pestle” was classified as an equipment. Information related to the overall objective of the protocol, applications, advantages, limitations and provenance was represented in our ontology; these data elements were validated by domain experts as they were mapping them to the ontology. Information about the sample (strain, line, genotype, developmental stage, organism part, growth conditions, treatment type and quantity used) was identified in published and unpublished protocols and could easily be mapped to the ontology.

Materials were also identified and mapped; interestingly, domain experts recognized different types of materials, for instance, instruments (including laboratory consumables), reagents, kits and software. In the resulting ontology we included “reagent” and “kit” under material entities; this made it easier for domain experts to identify terminology related to these classes. Published and unpublished protocols don’t differentiate across reagents, recipes, and kits; these are all usually listed under “Reagents”. However, domain experts reusing the protocols understand these under different categories. Reagents are understood as “ready to use”, often purchased; they also included mixtures prepared in the lab under reagents. Reagents are substances used in a chemical reaction to detect, measure, examine, or produce other substances [49]. Kits were considered as “gear consisting of a set of articles or tools for a specified purpose”. For instance, the Qiagen RNeasy Spin mini is a kit for purification of RNA from cells and tissues. However, a kit could also be an instrument; for instance, a digital recording transcribing kit, an instrument used to digitally record speech for transcription.

Recipes were identified as the most appropriate part of the protocol for including the details indicating how to prepare a particular solution, media, buffer, etc. The recipes could also describe how to make something; for example, “recipes describing how to clean laboratory equipment before starting the execution of a procedure”, see lab procedure 2 in our running example (Fig. 3 and Table 3); a recipe also is a way to include details regarding, e.g., the setup of HPLC separation methods. We classified the term “recipe” as a textual entity. The execution of a recipe was also considered, we included the term “recipe execution” as a planned process.

#### Competency questions

The RDF generated from instantiating the ontology was loaded in our SPARQL endpoint; the competency questions were then executed against this dataset. In general the expected information was retrieved; however, as domain experts were looking at the results, they started to reformulate the questions by asking for more information. For instance, domain experts asked for reagents to be linked to catalogs from the manufacturers or to resources like PubChem [50]. They were also interested in linking the samples/organisms to DBPEDIA [51] and NCBI taxonomy database [17, 18]; similarly, safety information was deemed as another case for establishing links between entities in the protocol and other information resources in the web. Some queries making use of linked data resources via federated queries illustrate this requirement; as additional information was necessary, we were looking into linked data resources that could complement the retrieved information. Queries like “Retrieve all the reagents and the information about where to buy them” illustrate how we were making use of other information resources; federated queries, see^2^, are retrieving complementary information from linked data resources such as DBpedia, Uniprot [52], PubChem, SNOMED over BioPortal and ChEBI. Some of the federated queries are presented in Table 4.

**Table 4:** Queries making use of external resources. Queries are available at https://smartprotocols.github.io/ queries/

Competency question	Was the question answered?	Other Information Resources	SPARQL	Comment
Retrieve all the protocols that use mouse as a sample	Yes. Could there be a short description about the organism and also, mouse is too specific, I may also be interested in rats and other rodents.	The DBPEDIA property dbo:order of includes individuals that belong to the order rodents, e.g. rats, hamsters, squirrels, etc. DBPEDIA also has dbo:abstract, this property allows us to retrieve information about rodents.	Query#1. Retrieve all the protocols with samples that belong to the Rodent order and also retrieve information for these samples	Additional information was useful but basic
Retrieve all the reagents used in the protocols	Yes. It is also useful to know where to buy these products.	PubChem has a list of vendors for some reagents. For instance, for sodium chloride it has more than ten vendors. Also, we are resolving the entities against the websites of the manufacturers.	Query #4.Retrieve all the reagents along with the different web sites to buy them and all the different manufacturers registered for every reagent	Additional information was useful
Retrieve the protocols in which Bromophenol blue is used	Yes. Could the applications for the reagent be included in the answer?	ChEBI is an external resource that has the applications for some reagents.	Query #23 Retrieve the protocols in which Bromophenol blue is used and tell me about the application of Bromophenol blue	Additional information was useful
Retrieve the steps that have CAUTIONS as alert messages from the protocol “X”	Yes. I would also like to have the diseases caused by this reagent	In this case we are making use of Bioportal and SNOMED (causative_agent_of).	Query #14. Retrieve all the diseases caused by the reagents in the protocol “Extraction of total RNA from fresh/frozen tissue (FT)”	Additional information was useful

## Applying the SMART protocols ontology to the definition of a minimal information model

**Fig. 4:**
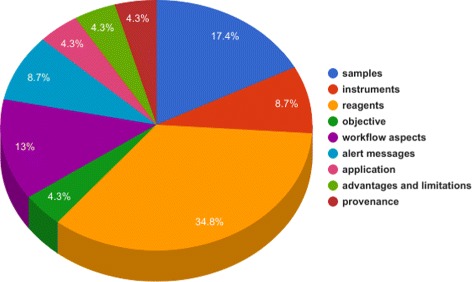
Distribution of SIRO elements

**Fig. 5:**
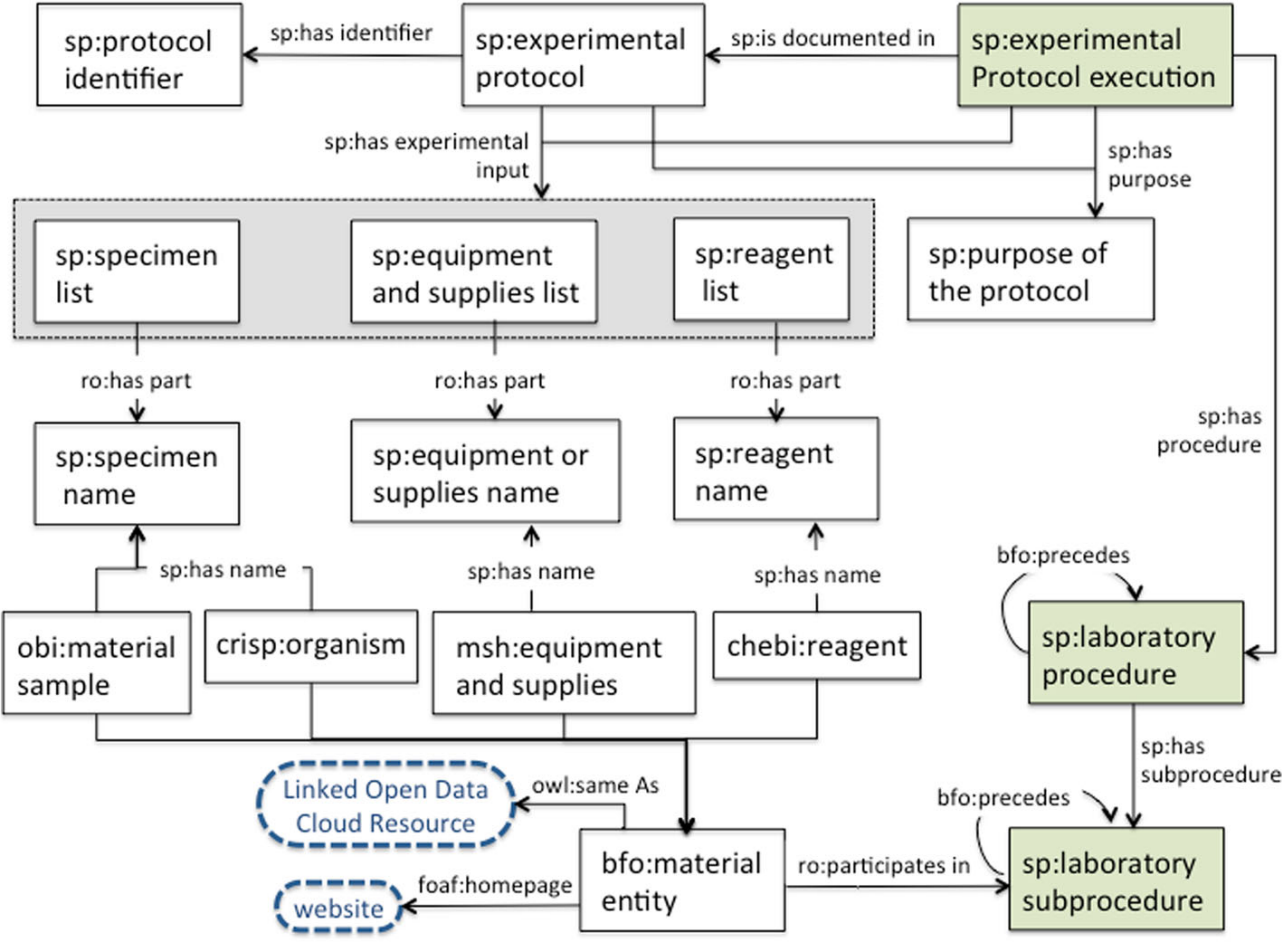
The SIRO model

Initially we developed the SP ontology and then the SIRO model. As we were representing the protocols as RDF we were also analyzing the competency questions; by doing so we saw a common pattern. From our competency questions, 17.4 percent were related to Samples, 8.7 percent were related to Instruments, 34.8 percent were related to Reagents Fig. 4. Furthermore, although the description of the workflow varies across our evaluation corpus, these data elements were always present. We focused on the manual identification of commonalities, the very minimal information shared across our corpus of documents. We then classified these data elements by mapping them to the SP ontology. This allowed us to determine higher abstractions to which the terminology could be mapped, e.g., “*sample*”, “*reagent*” and “*instrument*”. Domain experts discussed the granularity of the workflow description, whether the limitations of the protocol should or should not be reported, how to report the application of the protocol, etc. However, there was no disagreement about the need to report the objective of the protocol, e.g. “*method for the production of 3D cell lysates that does not compromise cell adhesion before cell lysis*”. Unlike samples, instruments and reagents, the objective is not always easily identifiable; it may be scattered throughout the document. It is, however, an important element; the description of the objective makes it easier for the readers to decide on the suitability of the protocol for their experimental problem. The SIRO model is illustrated in Fig. 5.

### The sample instrument reagent objective (SIRO) model

SIRO represents the minimal common information shared across experimental protocols. It serves two purposes. First, it extends available metadata for experimental protocols, e.g. author, title, date, journal, abstract, and other properties that are available for published experimental protocols. SIRO extends this layer of metadata by aggregating information about **S**ample, **I**nstrument, **R**eagent and **O**bjective –hence the name. Categories and instances of the data elements for SIRO are presented in Table 5. Second, SIRO makes it possible to frame and answer queries based on the minimal common data elements in experimental protocols. This facilitates finding specific protocols; if the owner of the protocol chooses not to expose the full content, as it is the case of publishers and/or laboratories, SIRO may be exposed without compromising the full content of the document. For instance, queries such as “*retrieve protocols that use samples from the rodent order*” or “*retrieve protocols that use Nucleic acid purification kits*” are executed using information that is also part of the SIRO model. Retrieving information related to steps, procedures, and recipes is only possible if the protocol is public, e.g. open access. In our case, CIAT facilitated some protocols for which only SIRO elements could be exposed; steps, alert messages and troubleshooting were considered as sensible information that should not be publically available.

**Table 5:** SIRO elements

Sample	Whole organism	Scientific name: Arabidopsis
		thaliana, Oriza sativa, mangifera
		indica, Mus musculus.
		Common names: Mousear Cress,
		rice, mango, mouse.
	Anatomical part	Leaf, stem, cells, tissues,
		membranes, organs, skeletal
		system, muscular system,
		nervous system, reproductive
		system, cardiovascular system, etc.
	Biomolecules	Nucleic acids: Deoxyribonucleic acid
		(DNA) and ribonucleic acid (RNA).
		Proteins: enzymes, structural or
		support proteins (keratin, elastin,
		collagen), antibodies, hormones, etc.
	Body fluids	Blood serum, saliva, semen, amniotic
		fluid, cerebrospinal fluid, gastric
		acid, etc.
Instrument	High-throughput	Liquid Handling Platforms, Real-Time
	equipment	PCR Detection System, Microplate
		Reader, etc.
	Instruments	Goggles, Bunsen burner, spot plate,
		pipet, forceps, test tube rack, mortar
		and pestle, etc.
	Laboratory glassware	Beaker, Erlenmeyer flask, graduated
		cylinder, volumetric flask, etc.
	Standard equipment	Balances, shakers, centrifuges,
		refrigerators, incubators,
		thermocyclers, fume hood, etc.
	Consumables	Weighing dishes, pipette tips, gloves,
		syringes, petri dishes, test tubes,
		micro centrifuge tubes, glass slides,
		filter paper, etc.
Reagents	Chemical	Glucose, ethanol, glycerol, chloroform,
	compound/Substance	acetic acid, isopropyl alcohol, etc.
	Solutions/buffers	70% ethanol, 10X PCR buffer,
		phenol:chloroform:isoamyl
		alcohol, etc.
	Cell culture media	Nutrient media, minimal media,
		selective media, differential media, etc.
Objective	Part of discourse	Here we present a detailed protocol
		for Smart-seq2 that allows the
		generation of full-length cDNA and
		sequencing libraries by using
		standard reagents

### Evaluating the SIRO model

For evaluating SIRO we extracted and populated the SIRO model with the RDF dataset that we used for the evaluation of the SP ontology. As the SIRO model does not expose the whole content of the protocol we also added five unpublished, private, protocols to the dataset. In total, for this evaluation we have 15 protocols in the SPARQL endpoint ^4^. For those queries involving instances of SIRO, we could satisfactorily retrieve the information required by the competency questions. Moreover, as SIRO complements bibliographic metadata information, the wealth of queries can be expanded. For instance:

- Retrieve the protocols and the list of reagents for documents authored by Yoshimi Umemura.
- Retrieve the protocols authored by Yoshimi Umemura and Beata Dedicova using rice leaves as sample.
- Retrieve the common reagents across the protocols “[Bio101] Subcutaneous Injection of Tumor Cells” and “Scratch Wound Healing Assay”.

## Discussion

### SMART protocols ontology

We propose the SP ontology to represent experimental protocols. It reuses the metadata structure, as well as some classes and properties, from OBI. It also builds upon experiences such as the BioAssay Ontology (BAO), The Experimental Factor Ontology (EFO), eagle-i resource ontology (ERO) and also the EXACT ontology. The SP Ontology also considers reporting structures such as ARRIVE, BRIDG as well as those from BioSharing. For representing “instruments”, “reagents/chemical compounds”, “organisms” and “sample/specimen” we reuse, amongst others, NCBI taxonomy, Cell Line Ontology (CLO) and Chemical Entities of Biological Interest (ChEBI). Our results indicate that the SP ontology makes it possible to represent all the data elements in the experimental protocols that we have analyzed.

### Modularization of the SP ontology

Modularization, as it has been implemented in SP, facilitates specializing the ontology with more precise formalisms. For instance, reagents, instruments and experimental procedures (actions), may be instantiated based on the activities carried out by a particular laboratory. We have two main modules in our ontology, the SP-Document and the SP-Workflow modules. The document module address issues related to archiving and representing the narrative. The workflow module aims to deliver a reusable executable object. In this way we make it possible for protocols to “*be born semantics*”. To “*be born semantics*” delivers a self-describing workflow embedded within a document from the onset. As a document, it is easily managed and understood by humans. As a self-describing workflow embedded within a document it is easily processed by machines. Our representation has some limitations with respect to machine processability; for instance, it is not suitable for robots to interpret it.

The document module facilitates archiving; publishers and laboratories can extend it depending on their use cases. The workflow module delivers an extensible representation describing the sequence of activities in an experimental protocol. Actions, as presented by [11], are important descriptors for biomedical protocols. However, in order for actions to be meaningful, attributes such as measurement units and material entities (e.g. sample, instrument, reagents, personnel involved) are also necessary. Our workflow representation makes it possible to link procedures and subprocedures to reagents, instruments, samples, recipes, hints, alert messages, etc. This is particularly useful because procedures and subprocedures can easily be reused and adapted; also, it allows researchers to retrieve very specific information and aggregate other data elements as it is needed. Formalizing workflows has an extensive history in Computer Science; not only in planning but also in execution -as in Process Life-cycle Management and Computer Assisted Design/Computer Assisted Manufacturing. The SP-workflow module helps to formalize the workflow implicit in protocols; our workflow specification has some limitations. For instance, loops, conditionals and other workflow constructs are currently being formalized as new use cases are identified. Our workflow constructs are easily extensible; we are also evaluating formal workflow languages for processes and adapting these to the biomedical scenario. Overcoming the limitations in the description of the workflow will make it possible to have an accurate representation of the protocol as an executable object for machines to fully process -including robots. The workflow nature implicit in experimental protocols should also be intelligible and manageable by humans; we are currently exposing the protocols in a format, RDF, that machines can understand for web purposes, e.g. discovery, interoperability.

### Limitations

Describing samples was particularly difficult because attributes like strain, line or genotype, developmental stage, organism part, growth conditions, age, gender, pre-treatment of the sample and volume/mass of sample, etc, are important depending on the experiment and the type of sample. Reagents and instruments were easier to describe as they only require the commercial name, manufacturer and identification number. However, linking reagents and instruments to other information resources is not as simple. Manufacturers don’t always offer Application Programing Interfaces (APIs) that make it possible to resolve these entities against their websites. For our experiment we had to scrape these websites in order to build the links. Furthermore, they don’t always use controlled vocabularies, common identifiers or describe chemicals in the same way; this made it difficult to search across their catalogs. Sigma-Aldrich and PubChem link to each other and PubChem has links to several manufacturers and vendors, this was deemed useful by domain experts. Linking was not initially considered by domain experts in their early competency questions; however, when they saw the answers for their queries, their expectation for linking data grew. In order to meet this demand, we re-formulated the queries by adding some external resources. This was received with satisfaction by domain experts; however, the expectation for more data continued growing. The use of external data sources was problem dependent, so were the external data sources to use.

### The SIRO model, application of the ontology

The SIRO model for minimal information breaks down the protocol in key elements that we have found to be common across our corpus of experimental protocols: *i)* Sample/ Specimen (**S**), *ii)* Instruments (**I**), *iii)* Reagents (**R**) and *iv)* Objective (**O**). Exposing SIRO makes it possible for laboratories and publishers to present key elements that frame questions often asked by researchers when searching for experimental protocols. SIRO was tested and results were satisfactory. External sources of information, e.g. vendor information from PubChem, can also be used to enrich SIRO elements. By extending the bibliographic metadata, SIRO is also extending the wealth of queries being supported; it provides specific information that is relevant to the description of the protocol.

## Conclusions

Experimental protocols are central to reproducibility and they are widely used in experimental laboratories. Our ontology and minimal information model have been validated with domain experts; our evaluations indicate that the SP ontology can represent experimental workflows and also that retrieving specific information from protocols represented with the SP ontology is possible. Both, the ontology and the SIRO model are easily adaptable. Experimental protocols describe step by step “*how to do or how to execute*” an experimental procedure. In our conceptualization experimental protocols have a document and a workflow component; as workflows embedded within documents, the experimental protocols should have complete information that allows anybody to recreate an experiment.

Our approach facilitates the generation of a self-describing document. It makes it possible to present meaningful information of experimental protocols without compromising the content. More importantly, it makes it possible to anchor information retrieval within a context that is meaningful for experimental researchers, e.g. reagents, samples and instruments participating in subprocedures. Queries such as *“What DNA extraction protocol is used on rice samples?”*, *“what amount of leaf tissue to use?”* are common for experimental researchers; answering these is possible with the SP ontology. In laboratory settings experimental protocols are usually managed just like any other document. However, these are plans for the execution of experiments; resources are allocated based on specifics described in the workflows of experimental protocols. The SMART Protocols approach generates a computable document that may interoperate with, for instance, inventories or Laboratory Information Management Systems (LIMS). Thus making it easier for researchers to plan according to available resources.

Harmonizing efforts such as EXACT, OBI, STAR [10], BRIDG and SMART Protocols ontology is important because without a clear semantics, reporting structure and a minimal information model for experimental protocols these will remain highly idiosyncratic. Moreover, without such consensus the experimental record will remain highly fragmented and therefore not easily processable by machines or reproducible by humans. Efforts such as the Resource Identification Initiative (RRId) [32, 53] and identifiers.org [54, 55] are central in the preservation of the experimental record; it is important that these efforts start to address reagents and instruments more broadly as these resources don’t always have identifiers. Being able to review the data makes it possible to evaluate whether the analysis and conclusions drawn are accurate. However, it does little to validate the quality and accuracy of the data itself. The data must be available, so does the experimental protocol detailing the methodology followed to derive the data. Journals and founders are now asking for datasets to be publicly available; there have been several efforts addressing the problem of data repositories; if data must be public and available, shouldn’t researchers be held to the same principle when it comes to methodologies? Openness and reproducibility are not only related to data availability; when replicating research, being able to follow the steps leading to the production of data is equally important.

The SP ontology is a digital object that follows the FAIR Principles [56]. Our ontology is **findable**; it is registered at Bioportal^5^, it is also available in github^6^ and the vocab.linkeddata.es^7^. The ontology is documented to facilitate the **reusability**; classes and object properties are documented with annotation properties imported from the OBI Minimal metadata. Reusing the ontology is easy as it has “preferred terms”, “definitions”, “definition sources”, “example of use”, “alternative terms”, etc; this makes it easier for others to know the context of the terminology as well as the suitability for addressing other use cases. The SP ontology was developed in OWL-DL and it is licensed under a Creative Commons Attribution 4.0 International License; in this sense SP ontology is **interoperable** and **accessible**.

## Endnotes

^1^ http://smartprotocols.linkeddata.es/sparql

^2^ https://smartprotocols.github.io/queries/

^3^ https://smartprotocols.github.io/annex/

^4^ https://smartprotocols.github.io/protocolsrdf/

^5^ http://bioportal.bioontology.org/ontologies/SP

^6^ https://smartprotocols.github.io/

^7^ http://vocab.linkeddata.es/SMARTProtocols/

## Abbreviations

BAO: The BioAssay ontology
BFO: The basic formal ontology
ChEBI: Chemical entities of biological interest
CIAT: Center for tropical agriculture
DAKA: Domain analysis and knowledge acquisition
DNA: Deoxyribonucleic acid
EFO: The experimental factor ontology
ERO: Eagle-i resource ontology
EXACT: The ontology of experimental actions
FT: Fresh tissue or frozen tissue
IAO: The information artifact ontology
LIMS: Laboratory information management
LISA: Linguistic and semantic analysis
MI: Minimal Information
NCBI taxonomy: The national center for biotechnology information taxonomy
NeOn methodology: Methodology for building ontology networks
OBI: The Ontology for biomedical investigations
OOPS: The ontology pitfall scanner
OWL-DL: Web ontology language based on description logic
PCR: The polymerase chain reaction
PICO model: Patient intervention comparison outcome
RNA: Ribonucleic acid
RO: The ontology of relations
SIRO model: Sample instrument reagent objective model
SMART Protocols: semantic representation for protocols
SP ontology: SMART protocols ontology
SP-Document: The document module of SMART protocols ontology
SP-Workflow: The workflow module of SMART protocols ontology
SPARQL: SPARQL protocol and RDF query language
